# A Case of Concealed Accidental Hemorrhage during the First Stage of Labor, with Recovery of the Mother under Conservative Treatment

**Published:** 1894-07

**Authors:** 


A CASE OF CONCEALED ACCIDENTAL HEMORRHAGE DURING THE FIRST STAGE OF LABOR, WITH RECOVERY OF THE MOTHER UNDER CONSERVATIVE TREATMENT.—Edward Reynolds, M.D. (*Bost. Med. and Surg. Journ.;* Vol. CXXX.; No. 12.) The patient, a primipara, aged thirty-eight, presented, at the commencement of labor, an elongated, rigid cervix with a patulous external os. Shortly after pains began the patient complained of feeling faint, and her pulse increased to 160. She rallied somewhat without treatment.

The next day she became jaundiced and began to look highly cachectic. Labor progressed very slowly, and on the second day her pulse was 110, skin extremely sallow, conjunctivæ yellow. She was feeble and apathetic, cachexia marked, and presented the appearance of a patient in advanced malignant disease. The uterus was small but very prominent, and in a state of tonic contraction with slight exacerbations. The child's vertex was presenting, the external os was soft and thin and about half dilated, the internal os was hard and rigid. The fetus was found to be dead and was extracted by forceps. The expression of the placenta was followed by a quart of dark old-looking clot. The patient's convalescence was uninterrupted. In the author's opinion, the death of the fetus was due to hemorrhage into the uterus during the first stage of labor, causing a separation of the placenta, as the mother felt active fetal movements just prior to the commencement of pains. The fetus was considerably macerated, but it is possibie for this condition to have occurred in forty-eight hours.—*Ex.*

